# Dimethyl 2,2′-[ethane-1,2-diylbis(sulfanedi­yl)]dibenzoate

**DOI:** 10.1107/S1600536810022403

**Published:** 2010-06-16

**Authors:** Xiaoming Hu, Jianghua Yu, Limin Yuan

**Affiliations:** a School of Chemistry and Chemical Engineering, Pingdingshan University, Pingdingshan 467002, People’s Republic of China; b College of Chemistry and Chemical Engineering, Yangzhou Universitry, Yangzhou 225002, People’s Republic of China

## Abstract

The title compound, C_18_H_18_O_4_S_2_, was synthesized by the reaction of 1,2-dibromo­ethane with methyl thio­salicylate. The complete molecule is generated by crystallographic twofold symmetry: two methyl benzoate units are linked by an –S–(CH_2_)_2_–S– bridging chain with a *gauche* S—CH_2_—CH_2_—S torsion angle [72.88 (16)°]. The two aromatic rings form a dihedral angle of 79.99 (6)°. In the crystal, adjacent mol­ecules are linked into a three-dimensional network by non-classical C—H⋯O hydrogen bonds.

## Related literature

For the potential use of dithiodibenzoates in the construction of diverse frameworks with tailored properties and functions, see: Humphrey *et al.* (2004 ▶); Li *et al.* (2007 ▶); Murugavel *et al.* (2001 ▶); Wang *et al.* (2004 ▶); Zhou *et al.* (2009 ▶).
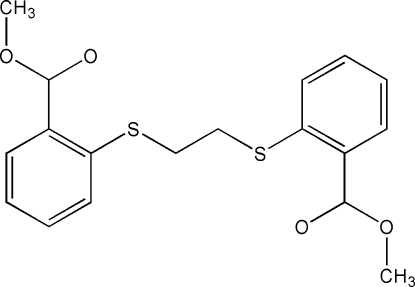


## Experimental

### 

#### Crystal data

C_18_H_18_O_4_S_2_
*M* *_r_* = 362.44
Monoclinic, 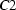

*a* = 15.077 (3) Å
*b* = 5.3913 (10) Å
*c* = 12.495 (2) Å
β = 120.662 (2)°
*V* = 873.6 (3) Å^3^
*Z* = 2
Mo *K*α radiation
μ = 0.32 mm^−1^
*T* = 296 K
0.52 × 0.32 × 0.22 mm

#### Data collection

Bruker SMART APEXII diffractometer
Absorption correction: multi-scan (*SADABS*; Bruker, 2006 ▶) *T*_min_ = 0.850, *T*_max_ = 0.932
3836 measured reflections
1948 independent reflections
1864 reflections with *I* > 2σ(*I*)
*R*_int_ = 0.023

#### Refinement

*R*[*F* ^2^ > 2σ(*F* ^2^)] = 0.032
*wR*(*F* ^2^) = 0.082
*S* = 1.08
1948 reflections
110 parameters
1 restraint
H-atom parameters constrained
Δρ_max_ = 0.23 e Å^−3^
Δρ_min_ = −0.15 e Å^−3^
Absolute structure: Flack (1983 ▶), 834 Friedel pairs
Flack parameter: −0.02 (7)

### 

Data collection: *APEX2* (Bruker, 2006 ▶); cell refinement: *SAINT* (Bruker, 2006 ▶); data reduction: *SAINT*; program(s) used to solve structure: *SHELXTL* (Sheldrick, 2008 ▶); program(s) used to refine structure: *SHELXTL*; molecular graphics: *DIAMOND* (Brandenburg, 2000 ▶); software used to prepare material for publication: *publCIF* (Westrip, 2010 ▶).

## Supplementary Material

Crystal structure: contains datablocks I, global. DOI: 10.1107/S1600536810022403/jh2166sup1.cif

Structure factors: contains datablocks I. DOI: 10.1107/S1600536810022403/jh2166Isup2.hkl

Additional supplementary materials:  crystallographic information; 3D view; checkCIF report

## Figures and Tables

**Table 1:** Hydrogen-bond geometry (Å, °)

*D*—H⋯*A*	*D*—H	H⋯*A*	*D*⋯*A*	*D*—H⋯*A*
C4—H4⋯O2^i^	0.93	2.51	3.435 (2)	171

Symmetry code: (i) 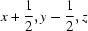.

**Fig. 1.**
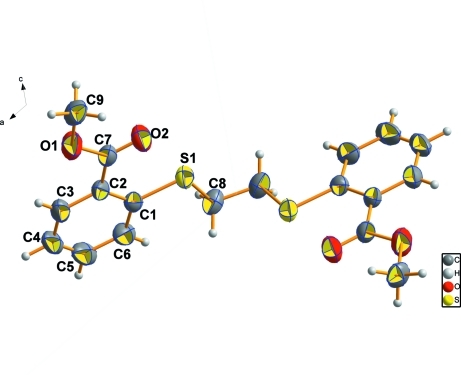
The molecular structure of (I), with atom labels and 50% probability displacement ellipsoids. Unlabeled atoms are related to labeled atoms by the symmetry code (-x, y, -z).

**Fig. 2.**
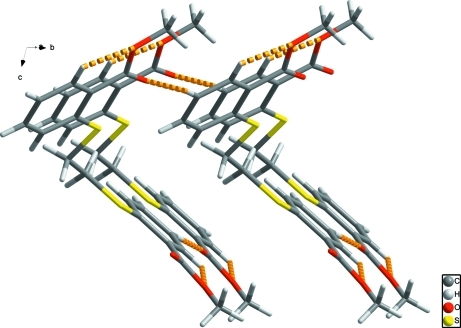
View of the connections inter- and intramolecules in (I) with the C—H···O interactions shown as orange dashed lines.

## supplementary crystallographic information

### Comment

The flexibility and conformational freedom of the disulfide derivatives of the benzoate – dithiodibenzoates - provides a possibility for the construction of diverse frameworks with tailored properties and functions (Murugavel *et al.*, 2001; Humphrey *et al.*, 2004; Wang *et al. *2004; Li *et al.*, 2007; Zhou *et al.*, 2009). The flexible bridging ligands adopt unusually twisted structures with different C—S—S—C torsion angles in constructing complexes.

The title compound described here is a longer analogue of 2,2'-dithiodibenzoate with the two methyl benzoate units interconnected by a flexible –S-(CH_2_)_2_-S– bridge (Fig. 1). The torsion angle S—CH_2_—CH_2_—S is 72.88 (16)°. The two aromatic rings form a dihedral angle of 79.99 (6)°. The C1(*sp*^2^)-S bond length [1.769 (2) Å] is significantly shorter than the C8(*sp*^3^)-S [1.814 (2) Å] bond length due to *p*-π conjugation. There are no significant S···S nor S···O contacts present in the structure and C—H···π (arene) hydrogen bonds and aromatic π···π stacking interactions are also absent. In the crystal, intermolecular C—H···O hydrogen bonds (Table 1) link the molecules into a three-dimensional supramolecular structure (Fig. 2).

### Experimental

The title compound was synthesized as follows: a solution of 1,2-dibromoethane (0.94 g, 5 mmol) in methanol (10 ml) was added dropwise to a mixture of methyl thiosalicylate (1.85 g, 11 mmol), KOH (0.617 g, 11 mmol) and ethanol (10 ml). The reaction mixture was stirred and heated for 12 h. The precipitate was filtered off, washed with water.Yield 72%; Colourless block crystals suitable for single-crystals X-ray analysis were obtained by recrystallization from an acetonitrile solution.

### Refinement

Hydrogen atoms were placed in calculated positions [C—H = 0.93–0.97 Å] and refined as riding [*U*_iso_(H) = 1.2Ueq(C) or 1.5Ueq(C_methyl_)].

### Crystal data

**Table:**

C_18_H_18_O_4_S_2_	*F*(000) = 380
*M**_r_* = 362.44	*D*_x_ = 1.378 Mg m^−^^3^
Monoclinic, *C*2	Mo *K*α radiation, λ = 0.71073 Å
Hall symbol: C 2y	Cell parameters from 2178 reflections
*a* = 15.077 (3) Å	θ = 2.7–27.4°
*b* = 5.3913 (10) Å	µ = 0.32 mm^−^^1^
*c* = 12.495 (2) Å	*T* = 296 K
β = 120.662 (2)°	Block, colourless
*V* = 873.6 (3) Å^3^	0.52 × 0.32 × 0.22 mm
*Z* = 2	

### Data collection

**Table:**

Bruker SMART APEXII diffractometer	1948 independent reflections
Radiation source: fine-focus sealed tube	1864 reflections with *I* > 2σ(*I*)
graphite	*R*_int_ = 0.023
ω scans	θ_max_ = 27.5°, θ_min_ = 1.9°
Absorption correction: multi-scan (*SADABS*; Bruker, 2006)	*h* = −19→18
*T*_min_ = 0.850, *T*_max_ = 0.932	*k* = −6→6
3836 measured reflections	*l* = −16→16

### Refinement

**Table:**

Refinement on *F*^2^	Secondary atom site location: difference Fourier map
Least-squares matrix: full	Hydrogen site location: inferred from neighbouring sites
*R*[*F*^2^ > 2σ(*F*^2^)] = 0.032	H-atom parameters constrained
*wR*(*F*^2^) = 0.082	*w* = 1/[σ^2^(*F*_o_^2^) + (0.0441*P*)^2^ + 0.1272*P*] where *P* = (*F*_o_^2^ + 2*F*_c_^2^)/3
*S* = 1.08	(Δ/σ)_max_ < 0.001
1948 reflections	Δρ_max_ = 0.23 e Å^−^^3^
110 parameters	Δρ_min_ = −0.15 e Å^−^^3^
1 restraint	Absolute structure: Flack (1983), 834 Friedel pairs
Primary atom site location: structure-invariant direct methods	Flack parameter: −0.02 (7)

### Special details

**Table:**

Geometry. All e.s.d.'s (except the e.s.d. in the dihedral angle between two l.s. planes) are estimated using the full covariance matrix. The cell e.s.d.'s are taken into account individually in the estimation of e.s.d.'s in distances, angles and torsion angles; correlations between e.s.d.'s in cell parameters are only used when they are defined by crystal symmetry. An approximate (isotropic) treatment of cell e.s.d.'s is used for estimating e.s.d.'s involving l.s. planes.
Refinement. Refinement of *F*^2^ against ALL reflections. The weighted *R*-factor *wR* and goodness of fit *S* are based on *F*^2^, conventional *R*-factors *R* are based on *F*, with *F* set to zero for negative *F*^2^. The threshold expression of *F*^2^ > σ(*F*^2^) is used only for calculating *R*-factors(gt) *etc*. and is not relevant to the choice of reflections for refinement. *R*-factors based on *F*^2^ are statistically about twice as large as those based on *F*, and *R*- factors based on ALL data will be even larger.

### Fractional atomic coordinates and isotropic or equivalent isotropic displacement parameters (Å^2^)

**Table:**

	*x*	*y*	*z*	*U*_iso_*/*U*_eq_	
C1	0.24609 (11)	0.0317 (4)	0.17607 (14)	0.0440 (3)	
C2	0.31876 (12)	0.1986 (4)	0.26461 (15)	0.0455 (4)	
C3	0.42223 (14)	0.1865 (5)	0.29514 (18)	0.0590 (5)	
H3	0.4700	0.2970	0.3530	0.071*	
C4	0.45387 (16)	0.0121 (6)	0.2402 (2)	0.0689 (7)	
H4	0.5224	0.0062	0.2606	0.083*	
C5	0.38363 (18)	−0.1516 (5)	0.1556 (2)	0.0694 (7)	
H5	0.4051	−0.2695	0.1192	0.083*	
C6	0.28109 (16)	−0.1440 (4)	0.12343 (19)	0.0577 (5)	
H6	0.2348	−0.2574	0.0660	0.069*	
C7	0.28831 (13)	0.3846 (4)	0.32683 (16)	0.0478 (4)	
C8	0.05804 (14)	−0.2095 (4)	0.02558 (17)	0.0525 (4)	
H8A	0.0717	−0.1916	−0.0420	0.063*	
H8B	0.0879	−0.3649	0.0679	0.063*	
C9	0.3458 (2)	0.6901 (7)	0.4813 (3)	0.0898 (8)	
H9A	0.2859	0.7821	0.4230	0.135*	
H9B	0.4035	0.8008	0.5233	0.135*	
H9C	0.3330	0.6119	0.5411	0.135*	
O1	0.36822 (11)	0.5052 (4)	0.41658 (15)	0.0793 (5)	
O2	0.20111 (11)	0.4248 (3)	0.30111 (15)	0.0675 (4)	
S1	0.11495 (3)	0.04719 (7)	0.13359 (4)	0.04790 (14)	

### Atomic displacement parameters (Å^2^)

**Table:**

	*U*^11^	*U*^22^	*U*^33^	*U*^12^	*U*^13^	*U*^23^
C1	0.0394 (7)	0.0510 (9)	0.0398 (7)	0.0110 (9)	0.0188 (6)	0.0069 (9)
C2	0.0355 (8)	0.0578 (11)	0.0388 (8)	0.0087 (7)	0.0157 (7)	0.0098 (7)
C3	0.0380 (9)	0.0805 (14)	0.0535 (10)	0.0051 (9)	0.0198 (8)	0.0137 (10)
C4	0.0461 (10)	0.100 (2)	0.0675 (12)	0.0304 (14)	0.0340 (9)	0.0303 (14)
C5	0.0659 (14)	0.0845 (17)	0.0697 (14)	0.0365 (12)	0.0433 (12)	0.0199 (12)
C6	0.0567 (11)	0.0623 (12)	0.0551 (11)	0.0187 (10)	0.0292 (9)	0.0041 (9)
C7	0.0387 (9)	0.0555 (11)	0.0409 (8)	−0.0024 (8)	0.0143 (7)	0.0010 (8)
C8	0.0529 (10)	0.0413 (9)	0.0474 (10)	0.0051 (8)	0.0141 (8)	−0.0028 (7)
C9	0.0861 (18)	0.0898 (19)	0.0753 (16)	−0.0203 (16)	0.0279 (14)	−0.0349 (16)
O1	0.0477 (7)	0.1046 (15)	0.0671 (8)	−0.0113 (9)	0.0159 (6)	−0.0323 (10)
O2	0.0418 (7)	0.0789 (10)	0.0765 (9)	−0.0027 (7)	0.0264 (7)	−0.0284 (8)
S1	0.03542 (19)	0.0520 (2)	0.0488 (2)	0.0033 (2)	0.01601 (15)	−0.0082 (2)

### Geometric parameters (Å, °)

**Table:**

C1—C6	1.401 (3)	C6—H6	0.9300
C1—C2	1.413 (3)	C7—O2	1.203 (2)
C1—S1	1.7685 (15)	C7—O1	1.324 (2)
C2—C3	1.406 (2)	C8—C8^i^	1.527 (4)
C2—C7	1.478 (3)	C8—S1	1.8143 (19)
C3—C4	1.384 (4)	C8—H8A	0.9700
C3—H3	0.9300	C8—H8B	0.9700
C4—C5	1.370 (4)	C9—O1	1.429 (3)
C4—H4	0.9300	C9—H9A	0.9600
C5—C6	1.386 (3)	C9—H9B	0.9600
C5—H5	0.9300	C9—H9C	0.9600
			
C6—C1—C2	118.08 (15)	O2—C7—O1	122.42 (19)
C6—C1—S1	121.47 (16)	O2—C7—C2	124.88 (16)
C2—C1—S1	120.44 (14)	O1—C7—C2	112.71 (16)
C3—C2—C1	119.52 (18)	C8^i^—C8—S1	108.52 (12)
C3—C2—C7	119.22 (18)	C8^i^—C8—H8A	110.0
C1—C2—C7	121.26 (14)	S1—C8—H8A	110.0
C4—C3—C2	120.8 (2)	C8^i^—C8—H8B	110.0
C4—C3—H3	119.6	S1—C8—H8B	110.0
C2—C3—H3	119.6	H8A—C8—H8B	108.4
C5—C4—C3	119.61 (18)	O1—C9—H9A	109.5
C5—C4—H4	120.2	O1—C9—H9B	109.5
C3—C4—H4	120.2	H9A—C9—H9B	109.5
C4—C5—C6	120.9 (2)	O1—C9—H9C	109.5
C4—C5—H5	119.5	H9A—C9—H9C	109.5
C6—C5—H5	119.5	H9B—C9—H9C	109.5
C5—C6—C1	121.0 (2)	C7—O1—C9	116.51 (17)
C5—C6—H6	119.5	C1—S1—C8	102.68 (10)
C1—C6—H6	119.5		
			
C6—C1—C2—C3	1.1 (3)	S1—C1—C6—C5	178.76 (16)
S1—C1—C2—C3	−178.69 (15)	C3—C2—C7—O2	173.83 (19)
C6—C1—C2—C7	−178.26 (18)	C1—C2—C7—O2	−6.8 (3)
S1—C1—C2—C7	1.9 (2)	C3—C2—C7—O1	−6.6 (3)
C1—C2—C3—C4	−0.4 (3)	C1—C2—C7—O1	172.80 (18)
C7—C2—C3—C4	178.97 (18)	O2—C7—O1—C9	−0.9 (3)
C2—C3—C4—C5	−0.4 (3)	C2—C7—O1—C9	179.6 (2)
C3—C4—C5—C6	0.5 (3)	C6—C1—S1—C8	2.60 (18)
C4—C5—C6—C1	0.3 (3)	C2—C1—S1—C8	−177.58 (15)
C2—C1—C6—C5	−1.1 (3)	C8^i^—C8—S1—C1	−176.60 (15)

Symmetry codes: (i) −*x*, *y*, −*z*.

### Hydrogen-bond geometry (Å, °)

**Table:**

*D*—H···*A*	*D*—H	H···*A*	*D*···*A*	*D*—H···*A*
C3—H3···O1	0.93	2.34	2.679 (3)	101
C4—H4···O2^ii^	0.93	2.51	3.435 (2)	171

Symmetry codes: (ii) *x*+1/2, *y*−1/2, *z*.
